# Structural and Secondary Electron Yield Properties of Titanium–Palladium Films with Laser-Treated Copper Substrate for Application in Neutron Generators

**DOI:** 10.3390/ma14051222

**Published:** 2021-03-05

**Authors:** Yong Gao, Sheng Wang, Jie Wang, Zhiming You, Jing Zhang, Yaocheng Hu, Yue Wu, Jiakun Fan, Haipeng Li, Qin Zhan, Hongguang Yang, Zhanglian Xu

**Affiliations:** 1 Shaanxi Engineering Research Center of Advanced Nuclear Energy & Shaanxi Key Laboratory of Advanced Nuclear Energy and Technology & School of Energy and Power Engineering, Xi’an Jiaotong University, Xi’an 710049, China; gaoyong1108@stu.xjtu.edu.cn (Y.G.); youzm19960311@stu.xjtu.edu.cn (Z.Y.); zhangjing1108@stu.xjtu.edu.cn (J.Z.); hyc1997@stu.xjtu.edu.cn (Y.H.); inak960119@stu.xjtu.edu.cn (J.F.); lihaipeng@xjtu.edu.cn (H.L.); 2 Department of Engineering Physics, Tsinghua University, Beijing 100084, China; y-wu20@mails.tsinghua.edu.cn; 3 Department of Reactor Engineering Research and Design, China Institute of Atomic Energy, Beijing 102413, China; zhanqin3891@126.com (Q.Z.); yanghg321@163.com (H.Y.)

**Keywords:** copper, laser-treated, neutron generator

## Abstract

Secondary electron emission (SEE) of the oxygen-free high-conductivity copper (OFHC) target surface in neutron generators limits the stability and improvement of the neutron yield. A novel-type target of titanium–palladium films coated on laser-treated OFHC target substrate was proposed and explored in this work to obtain low secondary electron yield (SEY) without introducing any components. The combination of Ti–Pd films and laser-treated OFHC substrate can effectively suppress secondary electron emission and enhance the adsorption ability to hydrogen isotopes with the existence of Pd film. The surface morphologies, surface chemical states, and SEYs of Ti–Pd films with laser-treated OFHC substrate were studied systematically for the first time. The XPS results showed that the laser-treated OFHC substrate surface was basically covered by Pd film. However, the Pd film surface was partially oxidized, with percentages of 21.31 and 10.02% for PdO and PdO_2_, respectively. The SEYs of Ti–Pd films with laser-treated OFHC substrate were all below 1 within the investigated primary energy range of 100–3000 eV, which would be sufficient for application in neutron generators. Specifically, the maximum SEY (*δ*_max_) of laser-treated OFHC substrate coated by Ti–Pd films was 0.87 with corresponding incident electron energy of 400 eV.

## 1. Introduction

With the advantages of light weight, transportability, safety, and adjustable neutron yield, neutron generators have been widely used in oil logging [1], neutron activation analysis (NAA) [2,3], neutron radiography [4,5,6], and boron neutron capture therapy (BNCT) [7,8]. Common neutron generators use ^2^H(d,n)^3^He (D–D) and ^2^H(t,n)^4^He (D–T) fusion reactions to produce neutrons with energies of 2.5 and 14.1 MeV, respectively [9]. The solid target D–D reaction commercial neutron generator produced by Adelphi has the highest neutron yield of 5 × 10^9^ n/s [10]. While, for the neutron generators using D–T reaction, the high neutron yield proposed and developed by Lanzhou University and Institute of Nuclear Energy Safety Technology [11,12] can reach to ~10^12^ n/s.

However, for some applications, such as BNCT and neutron radiography, higher neutron fluxes are required and neutron yield needs to be optimized to reduce irradiation time [13,14]. In addition, the service lifetime of the neutron generator should be extended. Since that almost bombarding ion energies are deposited in the target, the higher neutron yield results in a higher heat load on the target [15]. Therefore, the neutron yield is limited by the cooling ability of the target. Besides, the ion beam instability in the vacuum chamber of neutron generators is another problem that cannot be ignored when the neutron yield is increased. Beam instability is mainly induced by the secondary electron emission on the target, which affects the operation stability of the neutron generator, and poses a potential safety hazard [16]. In this case, characterizing these neutron generators is essential for improving their functionality. In-depth analysis of target cooling and secondary electron suppression is of significance for determining the neutron output and evaluating the machine behaviors at the steady state.

The neutron generator is mainly composed of an ion source, an ion extraction–accelerating system, a target system, and a vacuum system [16,17]. Generally, deuterium ions are produced in the ion source, then extracted and accelerated by the extraction–accelerating electrode. The ions then bombard the target, where D–D and D–T nuclear reactions occur and neutrons are generated. Due to the voltage difference, secondary electrons (SEs) are produced inevitably from the target surface when bombarded by the high-energy deuterium ion beam, and then accelerated back to the vacuum, as well as the ion source. This results in the formation of the secondary electron current, an increase in power loads, and the unstable operation of the neutron generator [16,18,19]. To reduce the secondary electron yield (SEY) in neutron generators, various methods of secondary electron suppression have been developed, such as Faraday cup suppression, magnetic and electric field suppression, and combinations of these methods [16,20]. However, the disadvantages are that they introduce extra components and occupy the neutron generation space. Faraday cup suppression provides a 70–80% reduction in the maximum SEY [21]. For the electric field suppression method, a resistor connecting the extraction–accelerating electrode to the target is applied to provide a bias voltage during the D+ ion bombardment. Secondary electrons can be completely suppressed with high resistance [16]. Different neutron generator structures require different bias voltages. However, too low or too high voltages can be detrimental. Too low a voltage may not be able to suppress the secondary electrons, while too high a voltage may suppress the secondary electrons, but it will result in a loss of the accelerating voltage, and then the ion energy will be reduced [21]. The magnetic field method is not as efficient as the electric field method, because the electric field changes the energy of the electrons, while the magnetic field only changes their direction [22].

Laser ablation is a novel way to produce a low-SEY surface, first proposed by Reza in 2014, to suppress the secondary electron emission in accelerators [23,24,25]. The secondary electrons emitted inside the porous structure are more likely to be trapped during collisions with the porous surface walls, resulting in a decrease in SEY. Specific to the application of the neutron generator, the porous target surface produced by laser ablation can also effectively suppress secondary electron emission without the introduction of any additional components [26]. Many works have been focusing on the principle and simulation of laser ablation to reduce SEY [27], and the accelerator tests on the laser-engineering surface are carried out [28]. However, the SEYs of film coating on laser-treated substrates are rarely mentioned before. For the first time, the influence of laser parameters on SEY was explored [24]. In addition, TiZrVHf film coating on laser-treated aluminum alloy and OFHC substrates was also studied [26,29].

Titanium film is widely used in neutron generator target systems as a getter for hydrogen and hydrogen isotopes (D_2_/T_2_) adsorption by forming titanium hydride [30,31,32]. By depositing palladium film on titanium film, the oxidation of titanium film can be prevented and the adsorption capacity of hydrogen isotopes can be improved [33,34,35,36]. Shugard et al. [37] found that the Pd coating facilitated hydrogen isotopes loading of titanium without the need for high-temperature vacuum activation, which means that the Ti–Pd bilayers are more easily loaded with D_2_/T_2_.

In this work, to further improve the neutron yield and reduce SEY in the neutron generator, Ti–Pd films were first proposed to coat the laser-treated oxygen-free high-conductivity copper (OFHC) substrate. This method has the advantages of effectively inhibiting secondary electron emission (SEE) and improving the adsorption capacity of Ti film to hydrogen isotopes, which can improve the neutron yield and neutron yield stability and prolong the service life of the neutron generators. The surface morphologies, surface average roughness, SEYs, and surface chemical compositions of Ti and Ti–Pd films coated on untreated and laser-treated OFHC substrates were explored.

## 2. Experiments and Methods

### 2.1. Sample Preparation

The OFHC substrates with purity of 99.95% and thickness of 500 µm were purchased from Heng Gong Machinery Materials Co., Ltd. (Dongguan, China). All samples were cut by a shearing machine and then polished; samples were cut to size 9 mm × 20 mm × 0.5 mm for SEY tests and to size 10 mm × 10 mm × 0.5 mm for X-ray photoelectron spectroscopy (XPS), laser scanning confocal microscopy (LSCM), scanning electron microscopy (SEM), and energy dispersion spectrum (EDS) tests. The OFHC substrates of samples #2 and #4 (Ti and Ti–Pd films with laser-treated substrates) were modified by a K20-CS nanosecond pulsed fiber laser (Han’s Laser, Shenzhen, China) to fabricate the micro- and nano-structure surface. The spot size, with a diameter of 15 μm, was focused on the surface, with pulse repetition frequency of 20 kHz, average power of 13.33 W, wavelength of 1064 nm, pitching spacing of 15 μm, and scanning speed of 20 mm/s. Before film deposition, untreated and laser-treated OFHC substrates were cleaned in an ultrasonic cleaning machine with acetone and absolute ethyl alcohol, each for 15 min. Ti and Ti–Pd films were deposited on the untreated and laser-treated OFHC substrates via a direct current (DC) sputtering method using argon as the sputtering gas. Ti and Pd cathode targets with a diameter of 3 inches were used with purities of 99.95 and 99.99%, respectively. For samples #3 and #4 (Ti–Pd films with untreated and laser-treated substrates, respectively), Pd film was deposited sequentially by rotating the sample disk to the bottom of the Pd target without exposure to air after Ti film deposition. The background pressure and working pressure during deposition in the vacuum chamber were about 5 × 10^−4^ Pa and 0.7 Pa, respectively. The discharge current during film deposition was about 0.44 A for Ti film and 0.52 A for Pd film, with the same discharge power of about 150 W, Ar gas flow of 22.4 sccm, and deposition temperature of about 35–37 °C for all samples. The coated samples were inevitably exposed to the atmosphere during the transfer, and there was also no sort of cleaning or baking prior to SEY tests. All information and results of the samples are shown in Table 1.

### 2.2. Characterization Methods

**Figure 1:**
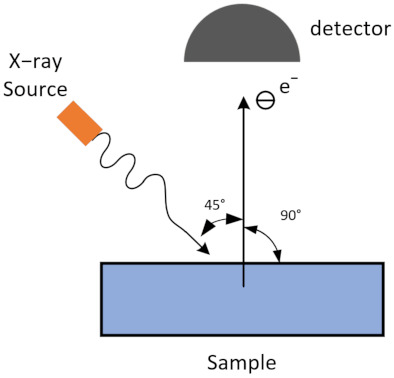
Schematic diagram of XPS testing.

The morphologies of the samples were characterized using a scanning electron microscope (SEM, TESCAN, Brno, Czech Republic) and Helios NanoLab 600 focused ion beam scanning electron microscope (FIB-SEM, FEI, Hillsboro, OR, USA). The surface roughness measurements for Ti and Ti–Pd films with untreated and laser-treated OFHC substrates were carried out via laser scanning confocal microscopy (LSCM, Olympus, Tokyo, Japan). X-ray photoelectron spectroscopy (XPS, Kratos, Manchester, UK) with a monochromatic Al K-alpha X-ray source was adopted to analyze the surface chemical properties of the samples. The angle relative to the target surface cross-sectioning these samples was 45° and the exit angle was 90° for the XPS scans, shown in Figure 1. SEY is defined as the ratio of secondary electron current (*I*_s_) to primary electron current (*I*_p_). The SEY properties were obtained using an SEY measurement device with an electron dose of 6.1 × 10^−6^ C·mm^−2^, primary electron continuous current of 10 nA, and test time of approximately 8 min. During SEY measurement, three identical samples for each case (#1–#4) prepared in different batches were tested. The test errors were within ±4.6, ±4.5, ±3.8, and ±4.4% for cases #1, #2, #3, and #4, respectively. According to the results of this study and our previous works, the repeatability of this method is very high, and the test error is always within ±5%. A detailed introduction of the SEY measurement device can be accessed in reference [24].

## 3. Results and Discussion

### 3.1. Surface and Cross-Section Morphologies

**Figure 2:**
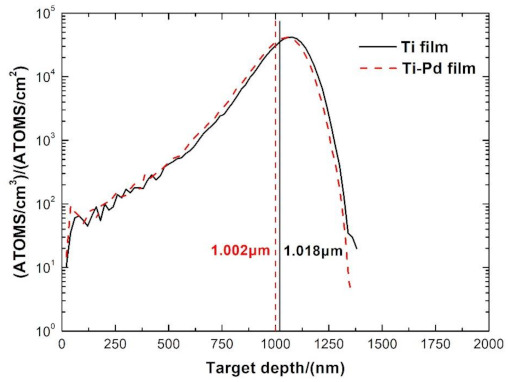
Simulation by Stopping and Range of Ions in Matter (SRIM) 2013 for 100,000 D^+^ penetration ranges with the energy of 150 keV in Ti film with a thickness of 2 μm and in Ti–Pd films with a thickness of 39.7 nm for the Pd film and 2 μm for the Ti film. The average D^+^ penetration range of Ti films and Ti–Pd films is 1.018 μm and 1.002 μm, respectively.

**Figure 3:**
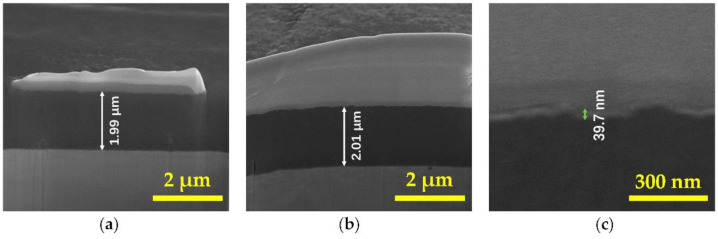
The film thicknesses of (**a**) Ti film, (**b**) the Ti layer of the Ti–Pd films, and (**c**) the Pd layer of the Ti–Pd films on untreated oxygen-free high-conductivity copper (OFHC) substrates.

Generally, the energy of the incident D^+^ ion beam in a neutron generator is about 150 keV with a corresponding mean penetration range of 1.018 μm in Ti film, calculated by Stopping and Range of Ions in Matter (SRIM) 2013 [38], as shown in Figure 2. To absorb as much D_2_ as possible into the Ti film, the Pd film should not be thick. Meanwhile, a decrease in Pd film thickness will reduce the adsorption ability of the D_2_ [35]. In this paper, the thicknesses of the Ti and Pd films were approximately 2.0 μm and 39.7 nm, respectively. As shown in Figure 2, the mean penetration range of D^+^ with energy of 150 keV in Ti–Pd films was 1.002 μm, which was quite close to that in Ti film, meaning that a very thin Pd film had little effect on the D^+^ penetration range. The exact thicknesses of the Ti and Ti–Pd films coated on untreated OFHC substrates are shown in Figure 3. Platinum (Pt) film was coated onto the Ti and Ti–Pd films with untreated and laser-treated OHFC substrates before they were cut by a focused gallium ion beam to reduce the damage to film samples.

**Figure 4:**
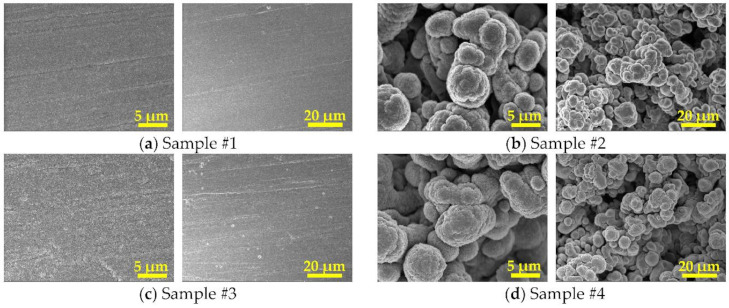
Surface morphologies of (**a**) sample #1—Ti film with untreated OFHC substrate, (**b**) sample #2—Ti film with laser-treated OFHC substrate, (**c**) sample #3—Ti–Pd films with untreated OFHC substrate, and (**d**) sample #4—Ti–Pd films with laser-treated OFHC substrate.

The surface morphologies of all samples are shown in Figure 4. The profile method was applied in this study to measure the surface average roughness for all samples via LSCM, while the average roughness (R*a*) was defined as the arithmetic mean of the absolute values of profile offset. Firstly, the laser source was used to scan the surface of the samples to obtain a two-dimensional image. Secondly, 10 different locations within the scan range were selected to perform the average roughness tests. Finally, the surface average roughness was obtained by averaging these results, since the sample surface with untreated or laser-treated OFHC substrate was relatively uniform. Compared with the surface average roughness of samples #1 and #3, those of sample #2 and #4 were much rougher after modification by a nanosecond pulse laser. Due to the reaction between the laser and the OFHC substrate, laser treatment induced enormous changes in the morphology and chemical composition of the OFHC surface, such as surface quenching, lattice change, and grain refinement with rapid melting and cooling [39]. The average roughness of samples #1, #2, #3, and #4 were 0.201, 7.502, 0.154, and 6.453 μm, respectively, as summarized in Table 1. Considering the corresponding value of untreated OFHC was 0.332 μm, the average roughness of sample #3 was a little bit lower than that of sample #1, and the average roughness of sample 1 was also lower than that of untreated OFHC, which may be caused by film deposition. In the process of film growth, higher temperature leads to faster molecular diffusion and migration, resulting in a decrease in the grain interval, and the roughness then decreases [40]. A similar trend was also found between samples #2 and #4.

**Figure 5:**
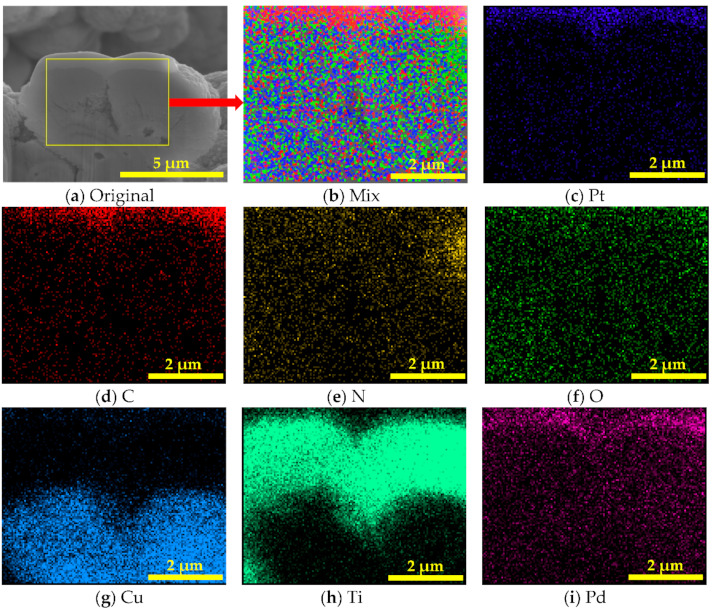
Cross-section mappings of chemical element distributions for Ti–Pd films with laser-treated OFHC substrate (sample #4) obtained from its energy dispersion spectrum. (**a**) Original, (**b**) Mix, (**c**) Pt, (**d**) C, (**e**) N, (**f**) O, (**g**) Cu, (**h**) Ti, (**i**) Pd.

Energy dispersion spectrum (EDS) element mappings of the Ti–Pd films with laser-treated OFHC substrate are shown in Figure 5. Elements O and N were found because the sample was laser processed in air, resulting in oxidation of the OFHC substrate. The C element was also introduced into the samples due to carbon impurity adhesion on the cut surface. Therefore, laser processing not only changed the surface morphology of the substrate but also induced a reaction between the copper substrate and gases in the air. It can also be seen that the OFHC substrate was basically covered by Ti and Pd films based on the mappings of Ti and Pd elements. A more quantitative investigation is given in the following section.

### 3.2. Surface Chemical Composition

XPS analysis was carried out to evaluate the chemical composition and states which play an essential role in secondary electron emission of Ti and Ti–Pd films. All XPS spectra were analyzed via CasaXPS software, using asymmetric Lorentzian (LA) line-shapes LA (1.1, 5, 7) for Ti metal and Gaussian (30%)–Lorentzian (70%) line-shapes for others, preceded by subtraction of the Shirley-type background [41].

**Figure 6:**
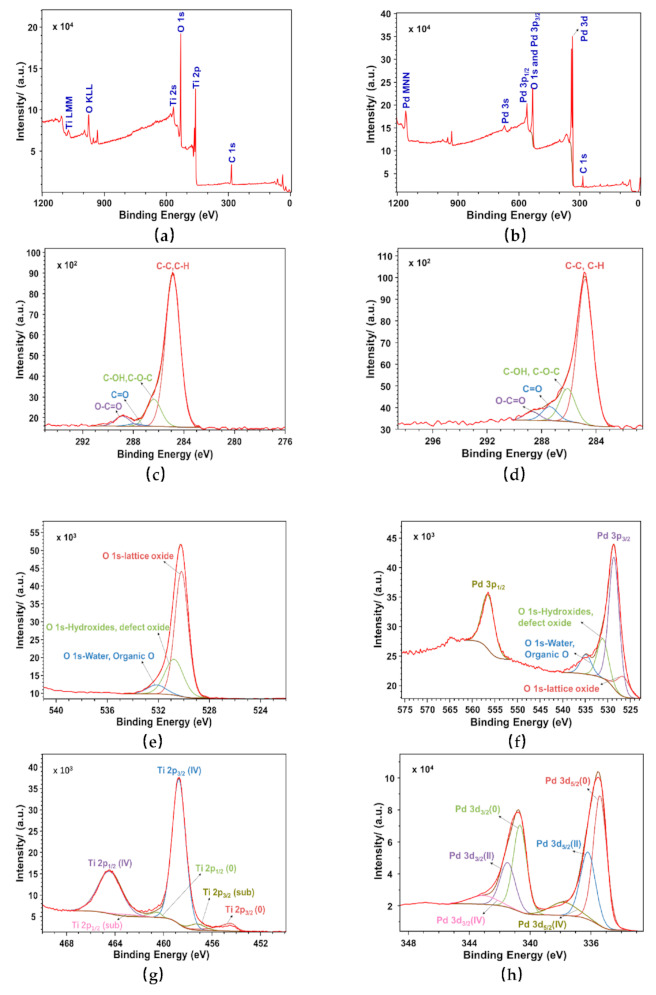
XPS spectra: (**a**) wide scan, (**c**) C 1s peak, (**e**) O 1s peak, and (**g**) Ti 2p peak for Ti film (sample #2) with laser-treated OFHC substrate, and (**b**) wide scan, (**d**) C 1s peak, (**f**) O 1s peak, and (**h**) Pd 3d peak for Ti–Pd films (sample #4) with laser-treated OFHC substrate.

The XPS results of Ti (sample #2) and Ti–Pd (sample #4) films with laser-treated OFHC substrates are shown in Figure 6 and summarized in Table 2, Table 3 and Table 4. In Figure 6a, it can be seen that the proportions of C, N, O, and Ti elements of sample #2 were 30.54, 0.15, 49.70, and 19.61%, respectively. The element ratios of C, N, O, and Pd elements in sample #4 were 19.22, 3.00, 52.27, and 25.51%, respectively, as shown in Figure 6b and Table 2. This indicated that the surface of sample #2 was basically covered by Ti film, while the surface of sample #4 was basically covered by Pd film. It can be speculated that C, N, and O elements were mostly introduced during sample transferal in the air [26]. As shown in Figure 6b, it can be seen that the O 1s peak and Pd 3p_3/2_ peak overlapped. In this case, the Pd 3p_1/2_ peak was used as a guide peak for fitting other peaks. The binding energy of C-C and C-H was about 284.8 eV [42]. As shown in Figure 6c and Table 3, the percentages of C-C/C-H, C-OH/C-O-C, C=O, and O-C=O in sample #2 were 79.27, 14.18, 1.28, and 5.27%, respectively, referring to carbon, alcohol, ketones, and acids and esters, respectively. The ratios of C-C/C-H, C-OH/C-O-C, C=O, and O-C=O in sample #4, shown in Figure 6d, were 72.20, 16.48, 6.92, and 4.40%, respectively. It could be inferred that the C element on the sample surface was mainly from the adsorption of amorphous carbon during sample transfer.

As shown in Figure 6e, the proportions of lattice oxide, hydroxides and defect oxides, and water and organic O in sample #2 were 88.15, 9.43, and 2.42%, respectively. As shown in Figure 6g, curve fitting of the Ti 2p spectrum showed that the binding energy levels of the three peaks for Ti metal, suboxide Ti, and TiO_2_ were at 454.4, 457.2, and 458.7 eV [29,43], with percentages of 7.11, 4.19, and 88.70%, respectively. This meant that the Ti metal of the sample #2 surface was almost fully oxidized. As shown in Figure 6f, the proportions of lattice oxide, hydroxides and defect oxides, and water and organic O on the surface of sample #4 were 27.53, 48.53, and 23.94%, respectively. In total, three peaks were exhibited at 335.4, 336.4, and 337.8 eV of the Pd 3d_5/2_ XPS spectrum, as shown in Figure 6h, which were attributed to bulk Pd, PdO, and PdO_2_ [44,45], corresponding to proportions of 68.67, 21.31, and 10.02%, respectively. The coated samples were exposed to the atmosphere before XPS tests. Therefore, the Pd film had been partially oxidized. Compared with the oxide percentage of 92.89% for the Ti film of sample #2, the oxide percentage was 31.33% for the Pd film of sample #4. The reason for this may be that Ti is more easily oxidized than Pd. All of these data are summarized in Table 4.

### 3.3. SEY

**Figure 7:**
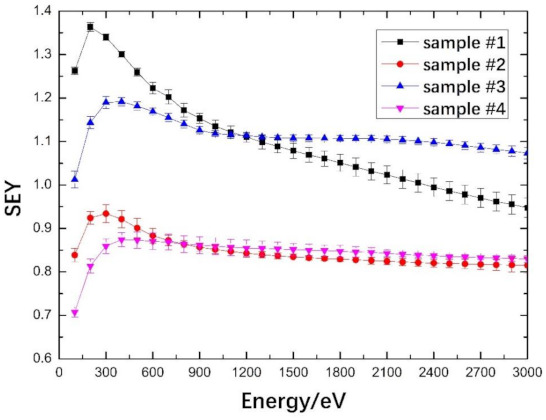
Secondary electron yield (SEY) curves of Ti and Ti–Pd films with untreated and laser-treated OFHC substrates. Sample #1—Ti film with untreated OFHC substrate, sample #2—Ti film with laser-treated OFHC substrate, sample #3—Ti–Pd films with untreated OFHC substrate, and sample #4—Ti–Pd films with laser-treated OFHC substrate.

SEY induced by electron bombardment is quite similar to the SEY induced by light ion bombardment [46]. Therefore, an electron source was applied to measure the SEY in this work. The SEY results of Ti and Ti–Pd films with untreated and laser-treated OFHC substrates are shown in Figure 7. Primary electrons with energy range of 100 to 3000 eV were used during the SEY measurements.

All the SEY curves increased at low electron energies, then decreased from no more than 400 eV, flattening at higher incident electron energies. The SEYs of sample #2 were always lower than those of sample #1 when the primary electron energy varied from 100 to 3000 eV. The maximum SEY (*δ*_max_) of sample #1 was 1.36 at primary energy (*E*_max_) of 200 eV. For sample #2, the *δ*_max_ was 0.93 with corresponding primary energy of 300 eV. Similar trends were also found between samples #3 and #4. The *δ*_max_ values of sample #3 and #4 were 1.19 and 0.87, respectively, corresponding to the same primary energy of 400 eV. The decrease in *δ*_max_ could be ascribed to the porous surfaces formed by laser processing. Porous structures can effectively reduce secondary electron emission because high-density micro- and nano-structures increase the probability of secondary electron capture stimulated from the surface, as described in the literature [23,24,47].

The *δ*_max_ values of Ti–Pd films with untreated or laser-treated OFHC substrate were slightly lower than those of Ti film, as shown in Figure 7. Lin et al. [48] studied the SEYs of fresh metals and found that the SEY of fresh Ti was slightly lower than that of Pd. However, under the same condition of air exposure, the Ti film was almost fully oxidized, with a TiO_2_ percentage of 88.70%. For Pd film, the total proportion of palladium oxide was 31.33%. The SEY of metal after air exposure is generally higher than that of the fresh metal [49]. In particular, TiO_2_ had much higher SEY than the fresh Ti, as reported [50]. These two aspects resulted in the maximum SEY of Ti–Pd films coated on laser-treated OFHC substrate being slightly lower than that of Ti film. For samples #1 and #3, SEYs of Ti film were lower than Ti–Pd films when the energies were higher than 1200 eV, this may be related to the penetration depth, which increases with the energy of the primary electron, while oxidation weakens with the depth. The SEYs induced by primary electrons with higher energies are closer to the fresh metal. However, SEYs of sample #1 were above 1 when the primary electron energies were lower than 2300 eV, while those of sample #3 were always higher than 1 in the investigated range. SEYs of these two samples were mostly higher than 1, indicating that they can cause the formation of electron cloud. When the primary electron energies were below 800 eV, the SEYs of sample #2 were higher than those of sample #4, while SEYs of sample #2 were lower than sample #4 when the primary electron energies were between 800 and 3000 eV. However, SEYs of samples #2 and #4 were always below 1 in the investigated range from 100 to 3000 eV, suggesting the entire suppression for secondary electron emission within the investigated range.

In addition, sample #4 had even lower SEYs, below 0.9, than sample #2 in the investigated electron energy range. Therefore, it can be reasonably speculated that the SEYs of sample #4 will always be below 0.9 at *E*_p_ ≥ 3000 eV as the curve levels off at higher energy. In addition, Pd film can prevent Ti film oxidation and improve the adsorption ability to hydrogen isotopes [33,34,35,36]. Therefore, Ti–Pd films with laser-treated OFHC substrate would be more effective for the design of neutron generator targets.

## 4. Conclusions

In this paper, a novel neutron generator target of Ti–Pd films coated on a laser-treated OFHC substrate was proposed. The SEYs, surface morphologies, surface average roughness, and chemical composition properties of the Ti and Ti–Pd films with untreated and laser-treated OFHC substrates were analyzed.

The SEM images showed that porous structures on the OFHC substrate were formed after laser processing, making them much rougher than the untreated ones. In the case of the same deposition parameters, adding Pd film coating on Ti film was able to reduce the average roughness, which possibly due to the increase in temperature leading to the acceleration of molecular diffusion and migration, resulting in the decrease in grain intervals and average roughness.

The surface of Ti–Pd films with a laser-treated OFHC substrate was composed of C, N, O, and Pd elements with the proportions of 19.22, 3.00, 52.27, and 25.51%. No Ti element was found. Therefore, it can be inferred that the surface was basically covered by Pd. The proportions of bulk Pd, PdO, and PdO_2_ were 68.67, 21.31, and 10.02%, respectively. It can be speculated that the partial oxidation of Pd metal was caused by air exposure during sample transfer before XPS tests.

The SEY curves of all the tested samples showed a similar trend: increasing at the beginning, reaching a peak value between 200 and 400 eV, and then decreasing at higher energy. The SEYs of Ti–Pd films were lower than those of Ti film with the same substrate. The δ_max_ of Ti–Pd films with laser-treated OFHC substrate was 0.87, with the corresponding incident electron energy of 400 eV. Sample #4 had very low SEYs in the range from 100 to 3000 eV. The laser ablation method can reduce maximum SEY below 1, indicating that there is no electron multiplication. Therefore, laser ablation, Faraday cup, magnetic field, and electric field methods can effectively suppress secondary electron emission, and the significant advantages of laser ablation method are not introducing any components and not increasing power load.

Cooling the target is also a major challenge in neutron generators. Compared with Ti and Ti–Pd films with untreated OFHC substrates, the surface roughness and surface area of Ti and Ti–Pd films with laser-treated OFHC substrates were obviously increased. As reported in the previous studies, the thermal conductivity of Ti film with laser-treated OFHC substrates was 3.5–11.5% higher than the one with untreated OFHC substrates measured from 25 to 300 °C [26]. It can be speculated that the thermal conductivity of Ti and Ti–Pd films with laser-treated OFHC substrates is also higher than those with untreated OFHC substrates. Combining with the results of this study, the low SEY and high thermal conductivity properties can be achieved by the combination of laser ablation technique and Ti and Ti–Pd films deposition, which is preferable for the application of neutron generator targets.

In summary, a low-SEY surface can be obtained by the combination of a laser-treated OFHC substrate and Ti–Pd films, which also improves neutron generator stability, enhances neutron yield, and increases adsorption ability to D_2_/T_2_. The results provide a novel idea for the design of neutron generator target. The secondary electron emission induced by D^+^ ion bombardment and the long-term stability of the Ti and Ti–Pd films with untreated and laser-treated OFHC substrates will be discussed in the future. Combined with laser ablation technique, titanium alloy films with strong absorption toward hydrogen isotopes will be explored as well.

## Figures and Tables

**Table 1:** Information of the Ti and Ti–Pd films with untreated and laser-treated oxygen-free high-conductivity copper (OFHC) substrates.

Sample	Film Coating	Substrate	*δ*_max_^1^	*E*_max_^2^/eV	R*a* ^3^/μm
# 1	Ti	Untreated OFHC	1.36	200	0.201
# 2	Ti	Laser-treated OFHC	0.93	300	7.502
# 3	Ti-Pd	Untreated OFHC	1.19	400	0.154
# 4	Ti-Pd	Laser-treated OFHC	0.87	400	6.543

^1^*δ*_max_ is the maximum secondary electron yield (SEY) within the investigated range. ^2^ ***E*_ma_**_x_ is the primary electron energy corresponding to the maximum SEY. ^3^ R*a* is the surface average roughness of the tested samples.

**Table 2:** Element compositions of Ti (sample #2) and Ti–Pd (sample #4) films with laser-treated OFHC substrates.

Sample	C	N	O	Ti	Pd
#2	30.54%	0.15%	49.70%	19.61%	0
#4	19.22%	3.00%	52.27%	0	25.51%

**Table 3:** Detailed proportions of C elements in Ti (sample #2) and Ti–Pd (sample #4) films with laser-treated OFHC substrates.

Sample	C-C/C-H	C-OH/C-O-C	C=O	O-C=O
#2	79.27%	14.18%	1.28%	5.27%
#4	72.20%	16.48%	6.92%	4.40%

**Table 4:** Detailed C and O element compositions and metal compounds of Ti (sample #2) and Ti–Pd (sample #4) films with laser-treated OFHC substrates.

Sample	O element			Metal	Metal Suboxide	Metal Oxide
	Lattice Oxide	Hydroxides/Defect Oxides	Water/Organic O			
#2	88.15%	9.43%	2.42%	Ti metal	Ti suboxide	TiO_2_
				7.11%	4.19%	88.70%
#4	27.53%	48.53%	23.94%	Pd metal	PdO	PdO_2_
				68.67%	21.31%	10.02%

## Data Availability

All data included in this study are available upon request by contact with the corresponding author.

## References

[1] Huang Z., Wang J., Ma Z., Lu X., Wei Z., Zhang S., Liu Y., Zhang Z., Zhang Y., Yao Z. (2018). Design of a compact D–D neutron generator. Nucl. Instrum. Methods Phys. Res. Sect. A-Accel. Spectrom. Dect. Assoc. Equip..

[2] Reijonen J., Leung K.-N., Firestone R.B., English J.A., Perry D.L., Smith A., Gicquel F., Sun M., Koivunoro H., Lou T.-P. (2004). First PGAA and NAA experimental results from a compact high intensity D–D neutron generator. Nucl. Instrum. Methods Phys. Res. Sect. A-Accel. Spectrom. Dect. Assoc. Equip..

[3] Marchese N., Cannuli A., Caccamo M.T., Pace C. (2017). New generation non-stationary portable neutron generators for biophysical applications of Neutron Activation Analysis. Biochim. Biophys. Acta-Gen. Subj..

[4] Bishnoi S., Sarkar P.S., Thomas R.G., Patel T., Pal M., Adhikari P.S., Sinha A., Saxena A., Gadkari S.C. (2019). Preliminary Experimentation of Fast Neutron Radiography with D-T Neutron Generator at BARC. J. Nondestruct. Eval..

[5] Wang J., Li Y., Wang Y., Li T., Zhang Z. (2019). Design and Optimization of a Fast Neutron Radiography System Based on a High-Intensity D-T Fusion Neutron Generator. Nucl. Technol..

[6] Bergaoui K., Reguigui N., Gary C.K., Cremer J.T., Vainionpaa J.H., Piestrup M.A. (2014). Design, testing and optimization of a neutron radiography system based on a Deuterium–Deuterium (D–D) neutron generator. J. Radioanal. Nucl. Chem..

[7] Sahoo G.S., Sharma S.D., Tripathy S.P., Bandyopadhyay T. (2017). Design and dosimetric evaluation of beam shaping assembly for BNCT of compact D–T neutron generator by Monte Carlo simulation. Biomed. Phys. Eng. Express.

[8] Kasesaz Y., Karimi M. (2016). A novel design of beam shaping assembly to use D-T neutron generator for BNCT. Appl. Radiat. Isot..

[9] Remetti R., Lepore L., Cherubini N. (2017). Development and experimental validation of a monte carlo modeling of the neutron emission from a dt generator. Nucl. Instrum. Methods Phys. Res. Sect. A-Accel. Spectrom. Dect. Assoc. Equip..

[10] Metwally W.A., Taqatqa O.A., Ballaith M.M., Chen A.X., Piestrup M.A. (2017). Neutron and photon dose mapping of a DD neutron generator. Radiat. Prot. Dosim..

[11] Song G., Wang G., Yu Q., Wang W., Chen C., Wu Y. (2015). Design and analyses of rotating tritium target system for high intensity D–T fusion neutron generator. J. Fusion Energy..

[12] Lu X., Wang J., Zhang Y., Li J., Xia L., Zhang J., Ding Y., Jiang B., Huang Z., Ma Z. (2016). Design of a high-current low-energy beam transport line for an intense DT/DD neutron generator. Nucl. Instrum. Methods Phys. Res. Sect. A-Accel. Spectrom. Dect. Assoc. Equip..

[13] Fantidis J., Antoniadis A. (2015). Optimization study for BNCT facility based on a DT neutron generator. Int. J. Radiat. Res..

[14] Taylor M., Sengbusch E., Seyfert C., Moll E., Radel R. (2017). Thermal neutron radiography using a high-flux compact neutron generator. Phys. Procedia..

[15] Kromer H., Adams R., Soubelet B., Zboray R., Prasser H.M. (2019). Thermal analysis, design, and testing of a rotating beam target for a compact DD fast neutron generator. Appl. Radiat. Isot..

[16] Huang Z., Bai X., Liu C., Wang J., Ma Z., Lu X., Wei Z., Zhang Z., Zhang Y., Yao Z. (2019). Study on secondary electron suppression in compact D–D neutron generator. Nucl. Sci. Tech..

[17] Qiao Y. (2008). Progress in studies and Applications of neutron tube. Nucl. Electron. Detect. Technol..

[18] Meng X., Dong Z. (2018). Dynamic Study on Effect of Secondary Electron on Ion Beam Quality. High Power Laser Part. Beams.

[19] Jin D. (1998). The Formation Process of the Electron Current in a Sealed Neutron Tube. Acta Sci. Nat. Univ. Jilin..

[20] Yang Z., Peng Y., Long J., Lan C., Dong P., Shi J. (2012). Suppression secondary electrons from target surface under pulsed ion beams bombardment. High Power Laser Part. Beams.

[21] Jin D.Z., Yang Z.H., Dai J.Y. (2009). Simulation for Suppressing of Secondary Electrons in Neutron Generator. J. Univ. Electron. Sci. Technol. China.

[22] Waltz C., Ayllon M., Becker T., Bernstein L., Leung K.N., Kirsch L., Renne P., Van Bibber K. (2017). Beam-induced back-streaming electron suppression analysis for an accelerator type neutron generator designed for ^40^Ar/^39^Ar geochronology. Appl. Radiat. Isot..

[23] Valizadeh R., Malyshev O.B., Wang S., Zolotovskaya S.A., Gillespie W.A., Abdolvand A. (2014). Low secondary electron yield engineered surface for electron cloud mitigation. Appl. Phys. Lett..

[24] Wang J., Gao Y., Fan J., You Z., Wang S., Xu Z. (2019). Study on the effect of laser parameters on the SEY of aluminum alloy. IEEE Trans. Nucl. Sci..

[25] Bajek D., Wackerow S., Zanin D.A., Baudin L., Bogdanowicz K., Valdivieso E.G.-T., Calatroni S., Girolamo B.D., Sitko M., Himmerlich M. (2020). Role of surface microgeometries on electron escape probability and secondary electron yield of metal surfaces. Sci. Rep..

[26] Wang J., Gao Y., You Z., Fan J., Zhang J., Yang S., Guo S., Wang S., Xu Z. (2019). Laser Processed Oxygen-Free High-Conductivity Copper with Ti and Ti–Zr–V–Hf Films Applied in Neutron Tube. Appl. Sci..

[27] Baudin L., Sitko M., Garion C., Chiggiato P., Delloro F., Gaslain F., Sennour M., Jeandin M., Bajek D., Wackerow S. (2019). Morphological and Chemical Characterization of Laser Treated Surface on Copper. Key Eng. Mater..

[28] Calatroni S., Valdivieso E.G.T., Neupert H., Nistor V., Fontenla A.T.P., Taborelli M., Chiggiato P., Malyshev O.B., Valizadeh R., Wackerow S. (2017). First accelerator test of vacuum components with laser-engineered surfaces for electron-cloud mitigation. Phys. Rev. Accel. Beams.

[29] Wang J., Gao Y., You Z., Fan J., Zhang J., Qiao Z., Wang S., Xu Z. (2019). Non-Evaporable Getter Ti-V-Hf-Zr Film Coating on Laser-Treated Aluminum Alloy Substrate for Electron Cloud Mitigation. Coatings.

[30] Williams D.L., Vainionpaa J.H., Jones G., Piestrup M.A., Gary C.K., Harris J.L., Fuller M.J., Cremer J.T., Ludewigt B.A., Kwan J.W. High Intensity, Pulsed, D-D Neutron Generator. Proceedings of the 20th International Conference on Application of Accelerators in Research and Industry.

[31] Bergaoui K., Reguigui N., Gary C.K., Brown C., Cremer J.T., Vainionpaa J.H., Piestrup M.A. (2014). Development of a new deuterium-deuterium (D-D) neutron generator for prompt gamma-ray neutron activation analysis. Appl. Radiat. Isot..

[32] Liu Y., Byrne P., Wang H., Koltick D., Zheng W., Nie L. (2014). A compact DD neutron generator-based NAA system to quantify manganese (Mn) in bone in vivo. Physiol. Meas..

[33] Benvenuti C., Chiggiato P., Cicoira F., L’Aminot Y., Ruzinov V. (2004). Vacuum properties of palladium thin film coatings. Vacuum.

[34] Wang J., Wang Y., Xu Y., Zhang B., Wei W. (2016). Research on the secondary electron yield of TiZrV-Pd thin film coatings. Vacuum.

[35] Lim H.R., Eom N.S.A., Cho J.-H., Cho H.-B., Choa Y.-H. (2018). Hydrogen gettering of titanium palladium/palladium nanocomposite films synthesized by cosputtering and vacuum-annealing. Int. J. Hydrog. Energy.

[36] Wang J., Zhang B., Xu Y., Wang Y. (2017). Research on deposition rate of TiZrV/Pd film by DC magnetron sputtering method. Nucl. Sci. Tech..

[37] Shugard A.D., Walters R.T., Blarigan P.V. (2012). Titanium tritide radioisotope heat source development: Palladium-coated titanium hydriding kinetics and tritium loading tests. Energy Conv. Manag..

[38] Ziegler J.F., Ziegler M.D., Biersack J.P. (2010). SRIM-The stopping and range of ions in matter (2010). Nucl. Instrum. Methods Phys. Res. Sect. B Beam Interact. Mater. Atoms.

[39] Boinovich L.B., Modin E.B., Sayfutdinova A.R., Emelyanenko K.A., Vasiliev A.L., Emelyanenko A.M. (2017). Combination of Functional Nanoengineering and Nanosecond Laser Texturing for Design of Superhydrophobic Aluminum Alloy with Exceptional Mechanical and Chemical Properties. ACS Nano.

[40] Wu X., Lai F., Lin L., Lin L., Qu Y. (2008). Influence of thickness on the structural and optical properties of vanadium oxide thin films. Chin. J. Lasers.

[41] Toyoshima R., Yoshida M., Monya Y., Kousa Y., Suzuki K., Abe H., Mun B.S., Mase K., Amemiya K., Kondoh H. (2012). In Situ Ambient Pressure XPS Study of CO Oxidation Reaction on Pd(111) Surfaces. J. Phys. Chem. C.

[42] Wang J., Sian T., Valizadeh R., Wang Y., Wang S. (2018). The Effect of Air Exposure on SEY and Surface Composition of Laser Treated Copper Applied in Accelerators. IEEE Trans. Nucl. Sci..

[43] Hashimoto S., Tanaka A. (2002). Alteration of Ti 2p XPS spectrum for titanium oxide by low-energy Ar ion bombardment. Surf. Interface Anal..

[44] Chastain J., King J.R.C. (1992). Handbook of X-ray photoelectron spectroscopy. Perkin-Elmer Corporation.

[45] Bukhtiyarov A.V., Prosvirin I.P., Bukhtiyarov V.I. (2016). XPS/STM study of model bimetallic Pd–Au/HOPG catalysts. Appl. Surf. Sci..

[46] Ohya K. (2003). Comparative study of target atomic number dependence of ion induced and electron induced secondary electron emission. Nucl. Instrum. Methods Phys. Res. Sect. B Beam Interact. Mater. Atoms.

[47] Wang J., Gao Y., You Z., Fan J., Zhang J., Wang S., Xu Z. (2019). Laser Induced Nano and Micro Structures of Molybdenum Surface Applied in Multistage Depressed Collector for Secondary Electron Suppression. Appl. Sci.-Basel.

[48] Lin Y., Joy D.C. (2005). A new examination of secondary electron yield data. Surf. Interface Anal..

[49] Hilleret N., Scheuerlein C., Taborelli M. (2003). The secondary-electron yield of air-exposed metal surfaces. Appl. Phys. A-Mater. Sci. Process..

[50] Iyasu T., Inoue M., Yoshikawa H., Shimizu R. (2006). Experimental studies of secondary electron emission of TiO_2_ and Ti. Jpn. J. Appl. Phys..

